# Use of Telehealth in Mental Health (MH) Services During and After COVID-19

**DOI:** 10.1007/s10597-021-00861-2

**Published:** 2021-06-24

**Authors:** Todd Molfenter, Thomasine Heitkamp, Ann A. Murphy, Stephanie Tapscott, Stephanie Behlman, Olivia J. Cody

**Affiliations:** 1 University of Wisconsin – Madison, 1513 University Ave., Madison, WI USA; 2 College of Nursing and Professional Disciplines, 400 Oxford Street, Grand Forks, ND 58202 USA; 3 Department of Psychiatric Rehabilitation and Counseling Professions, Rutgers, School of Health Professions, 675 Hoes Lane West, Piscataway, NJ 08854 USA; 4 Department of Health Policy and Management, Rollins School of Public Health, Emory University, 1518 Clifton Rd, Atlanta, GA 30322 USA

**Keywords:** Telehealth, Mental health, Behavioral health, Technology adoption

## Abstract

COVID-19 social distancing guidelines caused a rapid transition to telephone and video technologies for the delivery of mental health (MH) services. The study examined: (a) adoption of these technologies across the MH service continuum; (b) acceptance of these technologies; and (c) intention of providers to use these technologies following the pandemic based on a sample of 327 MH organizations from 22 states during May–August 2020. There was widespread use of technology, with greater than 69% of organizations reporting using telephone or video for most services. For all video services and just three telephone services, organizations reported significantly greater odds of intending to use technology to deliver services post-COVID-19. Use of video was seen as more desirable as compared to telephone. The overall perceived ease of use and usefulness for video-based services and certain telephone services provide a promising outlook for use of these services post the COVID-19 pandemic.

## Introduction

The World Health Organization’s declaration of the Public Health Emergency of International Concern (PHEIC) (World Health Organization, 2020), due to the spread of COVID-19, created immediate opportunities and challenges for mental health providers. The concerns included increased physical, mental, and psychological harm among our citizenry (Wang et al., 2020) and a greater need for mental health services. Recent data underscore the importance of triaging mental health care to people at greatest risk, including young adults, racial/ethnic minorities, essential workers, and unpaid caregivers (Czeisler et al., 2020). In conjunction with the need for increased services, physical distancing was needed to lessen the spread of the COVID-19 virus.

The PHEIC was an opportunity for mental health providers to revisit their models of emergency and disaster response. It included an immediate call to action among administrators to support clinicians’ professional development as they continued to provide individual, family, and group assessments/diagnosis and therapy to socially isolated clients. The challenge for administrators was to determine organizational and individual readiness to change and assess capacity to diversify their structures for delivering care at a distance while significant uncertainty existed (Waldeck et al., 2020). Social distancing mandates demanded expanded use of telehealth to deliver services safely while maintaining access, efficacy, and engagement.

Telehealth includes both video and telephone connections between the provider and client/patient. Mental health services providers may refer to this type of delivery as telemental health, telepsychiatry, teletherapy, or telepsychology. Of note, use of this technology to deliver services is not new. However, implementing telehealth has been gradual, with efforts beginning in the 1960s and 1970s. Early adopters were required to pay for expensive equipment. Additionally, some government-funded programs encouraged expanding access to telehealth, but that model of care delivery dissipated when funding was no longer available (Shore et al., 2020). Barnett and Huskamp (2020) found that early telehealth services were provided primarily in rural states, underserved counties, and among clinicians employed in publicly owned facilities. Nevertheless, telehealth has increased over time, albeit somewhat limited. This includes use of telehealth among substance use disorder treatment providers, especially in rural areas (Uscher-Pines et al., 2020).

Since the beginning of the pandemic, however, the use of telehealth has increased substantially (American Psychiatric Association, 2020). Clinicians responded to the PHEIC by moving their practice online. For example, a June 2020 study published by the American Psychological Association (2020) found that 75% of the respondents to a survey were solely providing remote services, including therapy by telephone, telehealth platforms, or videoconferencing. That same percentage felt confident in their use of telehealth. Professional associations have supported this shift by updating their web pages with a host of content about telehealth guidelines that existed in the public domain and began to expand access to information, including frequent updates on changing federal guidelines (American Psychiatric Association, 2020).

The advantages of telehealth are included extensively in commentaries and trainings on this topic (Edirippulige & Armfield, 2017; Moore & Munroe, 2020). They include increased access and availability to care, convenience for both consumers and providers, a decrease in no shows, an increase in consumer demand, an increase in affordable and useable technology platforms, reduction in the risk of coronavirus transmission, and the ability to view the person in their environment (Benavides-Vaello et al., 2013; Pruitt et al., 2014). Additionally, existing literature underscored the proven efficacy of telehealth delivery with outcomes similar to in-person therapy delivered in an office setting (Bashshur et al., 2016; Hilty et al., 2013; Langarizadeh et al., 2017).

The disadvantages of telehealth relate to concerns about billing practices, state licensing requirements that prevent work across state borders, and confidentiality and privacy concerns. Additional disadvantages include lack of bandwidth for access to technology in some homes and areas, negative impact on the therapeutic relationship, and problems treating certain populations using telehealth (American Telemedicine Association, 2020; Ramirez et al., 2020). Petersen et al. (2020) describe the limitation of the research on client and clinician satisfaction, noting clinicians have greater concern about use of telehealth than clients do, with both expressing concerns about efficacy, confidentiality, the impact on the therapeutic relationship, and technology concerns.

The PHEIC has provided an unprecedented need to apply telehealth services. Against the background of mixed preferences toward telehealth utilization, a deeper understanding is needed regarding how telehealth was applied during COVID-19 and treatment providers’ reactions towards telehealth use. The manuscript focuses on mental health providers’ comfort level in using telephone and video-based modalities, their readiness to use technology tools to deliver telehealth, and the projected use of telehealth beyond the COVID-19 PHEIC.

## Methods

### Data Collection

The survey link was distributed by email, and data collected May 15–August 31, 2020. The sample arose from individuals representing organizations in the Mental Health Technology Transfer Center’s (MHTTC’s) databases. The MHTTCs are Substance Abuse and Mental Health Services Administration (SAMHSA)-funded technical assistance centers whose purpose is to provide training and technical assistance to the mental health prevention, treatment, and recovery workforce in the United States. Four regional MHTTCs and four regions representing 22 states distributed this survey. Survey links were sent to respondents using an email script approved by the University of Wisconsin’s Institutional Review Board (IRB). Individuals sent the survey link were either administrators of mental health programs or mental health clinicians (e.g., counselors, social workers, psychologists, case managers, and psychiatrists).

### Survey Instruments

The survey included the following components and scales:

#### Organizational Location and Type

Locations included rural, small city, suburban, and urban. Organizational types included specialty behavior health and health systems.

#### Organizational Role

There were two categories of survey respondents: administrators and individuals who provide clinical services. These groupings were included because of their role in the adoption continuum. Administrators are often the decision-makers on whether telehealth will be offered as a service delivery option, and those who provide clinical services influence the ongoing daily use of telehealth for mental health services.

#### Use of Telehealth

(a) The use of telephone and video-based services was assessed for the following services: screening and assessment, case management, multi-disciplinary team-based services (e.g., Assertive Community Treatment), peer supports, group therapy, individual therapy, medication management, psycho-education, therapy services during partial hospitalization, and therapy sessions during residential treatment with a binary Yes/No response option; and b) The projected use of telephone and video services for each these services following COVID-19 was assessed asking respondents to what extent they plan to use telephone or video services beyond use to maintain COVID-19 safety measures, with response options of less than before, about the same, little more than before, or much more than before.

#### Organizational Readiness for Technology Use

The Organizational Readiness for Technology Use predictive tool developed by Gustafson et al. (2007) was applied to assess dimensions of organizational readiness for telephone and video technologies. The tool used a 5-point Likert scale with endpoints of Strongly Disagree and Strongly Agree. The inventory assessed reimbursement for the technology during and after COVID-19; billing expertise for the technology; information technology experts to support use of the technology; the ease of integrating the technology into workflow; having a clinical champion for the technology; having staff, facilities, and equipment in place to support the technology; leadership support; patient support; patient accessibility; technology affordability for patients; staff support; and staff training. These variables were assessed for telephone and video in general and not by each type of mental health service modality.

#### Technology Acceptance

The survey included scales from the Technology Acceptance Model (TAM) (Daniel et al., 2004; Venkatesh et al., 2003). This model measures Ease of Use and Perceived Usefulness. The Ease-of-Use scale assesses (a) if it is easy to get it to do what I want, (b) offering technology does not require a lot of effort, (c) easy to learn, and (d) easy to use. Perceived Usefulness assesses (a) enhances our effectiveness, (b) improves our performance, (c) increases our productivity, and (d) is useful. The scale’s questions for the Perceived Usefulness and Ease of Use Variables had a 5-point Likert scale with endpoints of Strongly Disagree and Strongly Agree. The Intent to Use was a different 4-category ordinal scale with the selections: Following COVID-19, do you anticipate use of telephone/video for the following services will be: (a) less than before COVID-19 (1), (b) About the same (2), (c) a little more than before (3), (d) much more than before (4) or (e) N/A do not provide service.

### Data Analysis

Frequency distribution statistics were used to describe survey response rate, organizational characteristics (setting and type), participant job roles, use of telephone and video technologies for different SUD services, and intent to use telephone and video technologies to deliver the different services post-COVID-19. Linear mixed-effects models (LMM) were conducted to determine if there were differences in intent to use telephonic and video technologies based on organizational location or setting and the survey respondent’s role. For this analysis, a composite measure of the intent to use telephone and video technologies was created by averaging the intent to use scores across the different mental health services for each technology. The Intent to Use Telephone and Video technologies for the different services was analyzed using generalized linear mixed-effects models (GLMM) comparing “More Use” and “Little More Use” of the technology post-COVID-19 to “About the Same” and “Little Less” post-COVID-19. The Organizational Readiness for Technology Adoption variables were analyzed by comparing the Organizational Readiness for Technology Use factor scores between Telephone and Video using generalized linear mixed-effects models (GLMM) to determine factor differences between the technologies. Lastly, the TAM data was analyzed by conducting a mediational analysis of the Perceived Ease of Use and Perceived Usefulness variables compared to the composite Intent of Use variables for telephone and video services using linear regression. The analyses were conducted using the lme4 package in R Studio.

## Results

Three hundred and twenty-seven organizations were represented in the survey. Surveys were distributed to 1790 organizations that provide mental health services for a return rate of 18%. The respondents’ job categories were 50.8% administrators and 49.2% clinical services providers (Table 1). No significant difference appeared in the Intent to Use telephone or video services post-COVID-19 between the administrators and individuals who provide treatment and recovery services (p = 0.16). There were also no significant differences based on setting or organizational type. All reference variables, including the Administrator’s category, had future intent to use, or support use of phone and video that was significantly different from 0.

**Table 1:** Organizational characteristics

Variable	Predictor	n	%	Future intent phone			Future intent video		
				Estimate	95% Cl	p-value	Estimate	95% Cl	p-value
Organizational setting	Reference category: Rural	101	30.9	2.78	2.58–2.97	a	3.10	2.91–3.28	a
	Small City	70	21.4	− 0.23	− 0.54–0.08	0.14	− 0.05	− 0.33–0.24	0.74
	Suburban	64	19.6	0.06	− 0.25–0.37	0.72	− 0.11	− 0.40–0.18	0.47
	Urban	92	28.1	0.05	− 0.24–0.33	0.76	0.02	− 0.25–0.29	0.88
Organizational type	Reference category: Specialty Behavioral health	254	77.7	2.76	2.64–2.89	a	3.10	2.98–3.22	A
	Health Systems	73	22.3	− 0.06	− 0.33–0.22	0.68	− 0.12	− 0.37–0.14	0.37
Job role	Reference category: Administrator	166	50.8	2.77	2.61–2.93	a	3.14	3.00–3.29	a
	Clinician	161	49.2	− 0.06	− 0.29–0.18	0.64	− 0.15	− 0.36–0.06	0.16

^a^All intercepts were significant (i.e., for each reference category the future intent to use phone/video was significantly different from 0)

**Fig. 1:**
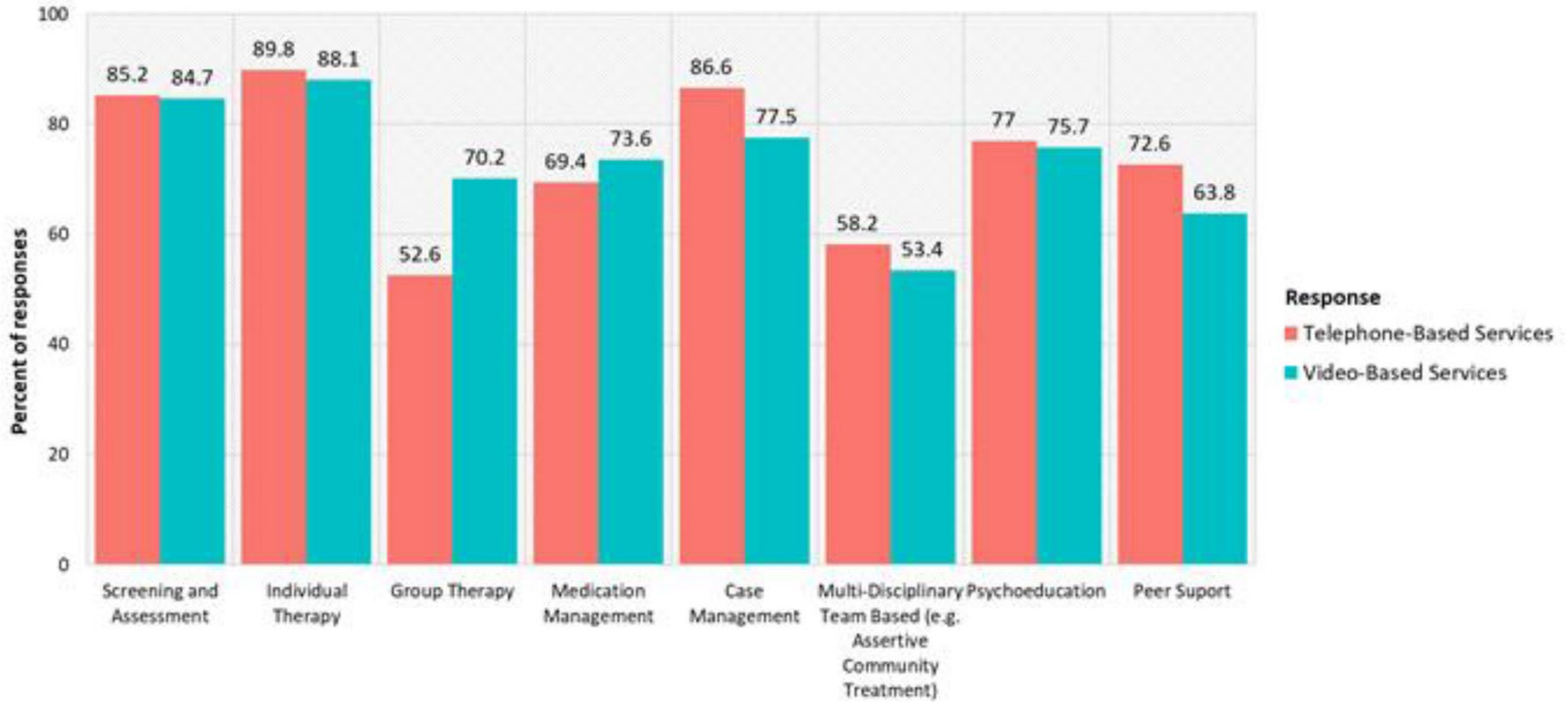
Current use of telehealth by service

The MH service that had the greatest percentage of telephone and video use was Individual Therapy at 89.8% for phone and 88.1% for video (Fig. 1). For the Intent to Use the technology, all the MH services had a positive odds ratio for wanting to use telephone or video technology “Much More” or a “Little More” following COVID-19 safety measures compared to using the technology the “About the Same” or a “Little Less” than before COVID-19 (Table 2). The odds ratios were not significant for using telephone for Group Therapy (p = 0.78), Medication Management (p = 0.18), or Multi-Disciplinary Team-Based (p = 0.20). All odds ratios were significant for video services, and video services were seen as more favorable than telephone for all services.

**Table 2:** Odds of using telehealth post-COVID-19 safety measures

	Telephone			Video			Video compared to telephone		
	Estimate	95% Cl	p-value	Estimate	95% Cl	p-value	Estimate	95% Cl	p-value
Screening and Assessment/Intake	2.10	1.32–3.34	0.002	7.10	3.93–12.82	< 0.001	3.39	2.11–5.44	< 0.001
Individual Therapy	2.97	1.63–5.42	< 0.001	14.26	6.37–31.94	< 0.001	4.79	2.81–8.18	< 0.001
Group Therapy	0.94	0.62–1.42	0.78	3.74	2.32–6.02	< 0.001	3.96	2.39–6.58	< 0.001
Medication Management	1.37	0.87–2.15	0.18	7.53	3.95–14.35	< 0.001	5.51	3.01–10.08	< 0.001
Case Management	2.34	1.36–4.02	0.002	5.23	2.79–9.78	< 0.001	2.24	1.35–3.72	0.002
Multi-disciplinary Team Based (e.g. Assertive Community Treatment)	1.54	0.80–2.97	0.20	4.31	2.04–9.10	< 0.001	2.80	1.50–5.23	0.001
Psychoeducation	1.98	1.03–3.83	0.041	8.37	3.68–19.00	< 0.001	4.22	2.35–7.55	< 0.001
Peer Support	2.43	1.35–4.36	0.003	5.42	2.76–10.66	< 0.001	2.23	1.31–3.81	0.003

Among the Organizational Readiness for Technology Use measures for telephone services, the factors that had the two highest ratings on the 5-point Likert scale were: (a) Telephonic/video counseling is affordable to patients (4.02, 95% Cl 3.89–4.14), and (b) Most of our patients can access telephonic/video technology (3.97, 95% Cl 3.84–4.10) (Table 3). For video services, the top two were: (a) our leadership supports the implementation of video counseling (4.10, 95% Cl 3.98–4.22), and (b) staff want video counseling to be sustained (4.03, 95% Cl 3.71–3.97). The significant differences between these technologies were telephone was seen as more advantageous for: (a) most of our patients can access telephonic technology (− 0.87, 95% Cl − 1.02–0.73, p < 0.001), (b) patients find telephonic counseling is easy to use (OR − 0.46, 95% Cl − 0.59–0.33, p < 0.001), and (c) telephonic counseling is affordable to patients (− 0.23, 95% Cl − 0.36–0.11, p < 0.001). Video was seen as more advantageous for (a) there is a clinical champion for the promotion of telephonic/video counseling (0.31, 95% Cl 0.18–0.44, p < 0.001) and (b) we anticipate being adequately reimbursed for the services we provide with telephonic/video counseling after COVID-19 (0.27, 95% Cl 0.14–0.20, p < 0.001).

**Table 3:** Organizational readiness for telephone and video use

	Telephone		Video		Video compared to telephone		
Question	Estimate	95% Cl	Estimate	95% Cl	Estimate	95% Cl	p-value
Our leadership supports the implementation of telephonic/ video counseling	3.96	3.84–4.08	4.10	3.98–4.22	0.14	0.03 to 0.25	0.011
There is a clinical champion for the promotion of telephonic/video counseling	3.32	3.18–3.46	3.63	3.49–3.77	0.31	0.18 to 0.44	< 0.001
Telephonic/video counseling is affordable to patients	4.02	3.89–4.14	3.78	3.66–3.91	− 0.23	− 0.36 to − 0.11	< 0.001
Most of our patients can access telephonic/video technology	3.97	3.84–4.10	3.10	2.97–3.23	− 0.87	− 1.02 to − 0.73	< 0.001
Patients find telephonic/video counseling is easy to use	3.93	3.80–4.05	3.47	3.34–3.59	− 0.46	− 0.59 to − 0.33	< .001
Patients want telephonic/video counseling to be sustained	3.83	3.70–3.96	3.75	3.62–3.87	− 0.08	− 0.20 to 0.03	0.16
Staff has been properly trained in telephonic/video counseling	3.66	3.54–3.79	3.68	3.55–3.80	0.01	− 0.10 to 0.13	0.82
Staff, facilities, and equipment, job descriptions, policies, are in place for sustaining telephonic/video counseling	3.60	3.46–3.73	3.65	3.51–3.78	0.05	− 0.08 to 0.18	0.43
Staff want telephonic/video counseling to be sustained	3.84	3.71–3.97	4.03	3.91–4.16	0.19	0.07 to 0.31	0.001
Telephonic/video counseling easily integrates into our workflow	3.80	3.68–3.92	3.85	3.72–3.97	0.05	− 0.07 to 0.16	0.43
We are adequately reimbursed for the services we provide with telephonic/video counseling during COVID-19	3.46	3.32–3.59	3.66	3.53–3.80	0.20	0.08 to 0.33	0.001
We anticipate being adequately reimbursed for the services we provide with telephonic/video counseling *after* COVID-19	3.18	3.04–3.32	3.45	3.31–3.59	0.27	0.14 to 0.40	< 0.001
We have the billing expertise to support use of telephonic/video counseling in our organization	3.71	3.58–3.84	3.82	3.69–3.95	0.11	− 0.01 to 0.22	0.071
We have the information technology expertise to support the use of telephonic/video counseling in our organization	3.76	3.63–3.89	3.80	3.67–3.93	0.04	− 0.08 to 0.16	0.55

**Fig. 2:**
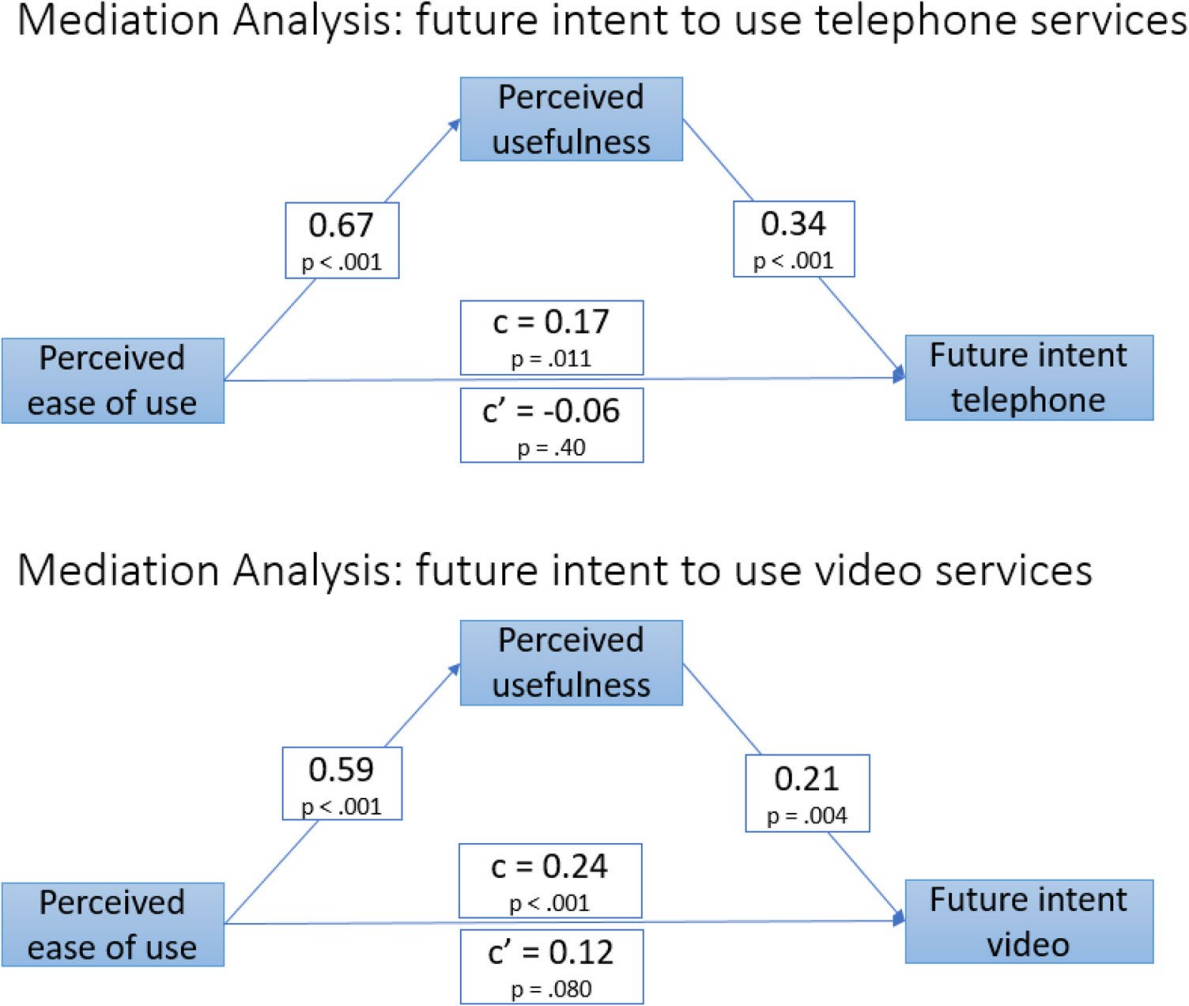
Technology acceptance model for telephone/video SUD services

The TAM posits that if a technology is Easy to Use and has Perceived Usefulness, it will lead to Intent to Use, resulting in actual use (Al-Emran et al., 2018; Szajna, 1996; Venkatesh & Bala, 2008). When this model is working as hypothesized and validated, Perceived Usefulness will mediate the relationship between Perceived Ease of Use and Intent to Use. Within the mediational analysis for telephone, Perceived Ease of Use was significantly associated with Future Intent (p = 0.011) (Fig. 2). When Perceived Usefulness was added to the equation, there was a significant relationship between Perceived Ease of Use and Perceived Usefulness (p < 0.001), between Perceived Usefulness and Future Intent (p < 0.001), and the relationship between Perceived Ease of Use and Future Intent was no longer significant (p = 0.40). This signifies complete mediation. A similar set of relationships were found in the mediational analysis for intent to use video services with the path between Perceived Ease of Use and Future Intent significant at p = 0.003 and not significant (p = 0.067) when Perceived Usefulness was added to the model (Fig. 2).

## Discussion

While telemental health has been in use for several decades, there has not been widespread, nationwide reliance on telehealth services within the mental health system until the recent changes prompted by the COVID-19 pandemic. In a very brief time span, most mental health services that could be provided remotely began to be delivered via telemental health. This study’s results, representing various regions across the country, demonstrate that the majority of respondents were providing a wide array of mental health services via telephone or video conferencing. Those services that involve one clinician and one client, including individual therapy and screening and assessment, were being delivered by the greatest number of respondents. Services delivered by multi-disciplinary teams (e.g., Assertive Community Treatment) or involving multiple clients (e.g., group therapy) were more challenging to deliver remotely and were less likely to be offered via telehealth.

While the transition to telemental health has been challenging for some (Murphy et al., 2020), this survey supports that organizations and clinicians are likely to continue using telehealth after the pandemic has passed. There was a preference for the use of video-based telehealth over phone-based telehealth for the delivery of services. That being said, there was also support for the continued use of telephone services for all modalities except group therapy, medication management, and multi-disciplinary team-based services. Telephone services are recognized as more affordable, easier to use, and accessible for clients. The perceived usefulness of telephone and video services mediated the relationship between the perceived ease of use and the intent to use telehealth in the future.

The survey findings support the continued use of telehealth services offered by mental health providers and organizations, as respondents indicated a desire to use these services more following the pandemic. Results suggest that clinic leadership and staff support the implementation and sustainment of telephone and video counseling. A review or update of telehealth reimbursement guidelines and associated regulations may be warranted to support providers and organizations in these efforts. The survey results also highlight factors that mental health providers and organizations believe allow for increased patient accessibility; specifically, telephone services are affordable and easily accessible. As mental health providers and organizations seek to incorporate patient-centered approaches, telehealth services may allow for greater patient choice in seeking and receiving services. Although findings from this survey indicate video-based telehealth services are viewed more favorably than telephone-based services, additional research is needed to identify which service modality can be delivered the most effectively. Similarly, because of its perceived patient ease of use and accessibility, telephone delivery may be supported for some clients. Hence, a diverse mix of service delivery modalities, including in-person services, may be needed to maximize flexibility, outcomes, and patient preferences.

The survey findings should be understood in the context of at least three limitations that affect generalizability. First, the mental health providers and organizations responding to the survey were from a convenience sample pulled from four regional MHTTC databases representing 22 states. Second, the return rate for this survey was 18%. Third, the survey’s email distribution was the only recruitment modality used and could have limited reach and response had multiple distribution approaches been used.

Previous research on telemental health has established that it can be effective, improve client satisfaction, and reduce the overall cost of care (Hilty et al., 2013; Langarizadeh et al., 2017). However, there has been some reluctance to implement telehealth more fully throughout the mental health system. This hesitation has been attributed, in part, to challenges regarding the technological skills of clinicians and clients, training needs for clinicians, financial investment in equipment, insurance coverage for services, and regulatory and compliance concerns (Langarizadeh et al., 2017; Mace et al., 2018). The pressure created by the pandemic, the temporary loosening of regulatory restrictions, and the expansion of insurance reimbursement has facilitated the expansion of these services. In 2018, 47% of survey respondents reported using telemental health (Mace et al., 2018). Just two years later, more than 89% were using telemental health for individual therapy in this study, resulting in a 42% increase in use. It is still unclear if the current telemental health services are being delivered with efficacy as those previously studied. This will have to be evaluated in the future.

In summary, the results demonstrate a large shift in telehealth use and provide an encouraging outlook for the use of telephone and video-based services after the COVID-19 pandemic. Future studies should continue to review the acceptance of these different service delivery approaches and their impact on care outcomes. In particular, integrating in-person, telephone, and video-based services to make them more patient-centered and achieve optimal outcomes should be studied in practice and research.
